# HIV-1-encoded antisense RNA suppresses viral replication for a prolonged period

**DOI:** 10.1186/1742-4690-9-38

**Published:** 2012-05-08

**Authors:** Mie Kobayashi-Ishihara, Makoto Yamagishi, Takuma Hara, Yuka Matsuda, Ryutaro Takahashi, Ariko Miyake, Kazumi Nakano, Tadanori Yamochi, Takaomi Ishida, Toshiki Watanabe

**Affiliations:** 1 Laboratory of Tumor Cell Biology, Department of Medical Genome Sciences, Graduate School of Frontier Sciences, The University of Tokyo, 4-6-1 Shirokanedai, Minato-ku, Tokyo, 1088639, Japan; 2 Department of Microbiology, Institute of Health Biosciences, University of Tokushima Graduate School, Kuramoto-cho, Tokushima, 7708503, Japan; 3 Research Center for Asian Infectious Disease, Institute of Medical Science, The University of Tokyo, 4-6-1 Shirokanedai, Minato-ku, Tokyo, 1088639, Japan

## Abstract

**Background:**

Recent evidence proposes a novel concept that mammalian natural antisense RNAs play important roles in cellular homeostasis by regulating the expression of several genes. Identification and characterization of retroviral antisense RNA would provide new insights into mechanisms of replication and pathogenesis. HIV-1 encoded-antisense RNAs have been reported, although their structures and functions remain to be studied. We have tried to identify and characterize antisense RNAs of HIV-1 and their function in viral infection.

**Results:**

Characterization of transcripts of HEK293T cells that were transiently transfected with an expression plasmid with HIV-1_NL4–3_ DNA in the antisense orientation showed that various antisense transcripts can be expressed. By screening and characterizing antisense RNAs in HIV-1_NL4–3_-infected cells, we defined the primary structure of a major form of HIV-1 antisense RNAs, which corresponds to a variant of previously reported *ASP* mRNA. This 2.6 kb RNA was transcribed from the U3 region of the 3′ LTR and terminated at the *env* region in acutely or chronically infected cell lines and acutely infected human peripheral blood mononuclear cells. Reporter assays clearly demonstrated that the HIV-1 LTR harbours promoter activity in the reverse orientation. Mutation analyses suggested the involvement of NF-κΒ binding sites in the regulation of antisense transcription. The antisense RNA was localized in the nuclei of the infected cells. The expression of this antisense RNA suppressed HIV-1 replication for more than one month. Furthermore, the specific knockdown of this antisense RNA enhanced HIV-1 gene expression and replication.

**Conclusions:**

The results of the present study identified an accurate structure of the major form of antisense RNAs expressed from the HIV-1_NL4–3_ provirus and demonstrated its nuclear localization. Functional studies collectively demonstrated a new role of the antisense RNA in viral replication. Thus, we suggest a novel viral mechanism that self-limits HIV-1 replication and provides new insight into the viral life cycle.

## Background

The genome of HIV-1 is about 9 kb with complex pathogenic mechanisms. HIV-1 encodes nine viral proteins, which have multiple functions in molecular events such as entry, integration, and viral gene expression, as well as the regulation of host molecular processes [1-3]. However, there still remain unanswered questions about the mechanisms of HIV-1 infection and pathogenesis despite advances in the knowledge of many diverse viral functions. For example, mechanisms for viral latency and reactivation have not been fully elucidated, and several events have been suggested to be involved in viral latency, including epigenetic reprogramming and modulated expressions of host factors [4-6].

Several researchers have embarked on studies to identify HIV-1 antisense RNAs (asRNAs) [7-11]. Using computational analysis, Miller predicted the existence of a novel gene in the antisense strand of HIV-1, which encodes ASP by a well-conserved open reading frame among many strains of HIV-1 [7]. Subsequently, the *ASP* mRNA was identified as a 2242 bp transcript covering the region between nucleotide positions 9608 and 7367 of the HXB2 strain in acutely infected A3.01 cells [8]. However, the primary structure and functions of HIV-1 asRNAs have not been fully clarified, although many researchers have proposed the potential importance of the asRNAs [9,12-20].

Other retroviral asRNAs have also been studied. In HTLV-1-infected T-cells, the *HBZ* RNA is expressed from the antisense strand of the HTLV-1 provirus. HBZ has been reported to be involved in the regulation of sense transcription and leukemogenesis by HTLV-1 [21-26]. Furthermore, feline immunodeficiency virus and Friend and Moloney murine leukemia virus have also been suggested to express antisense transcripts [27,28]. In addition, Ty1 retrotransposon was shown to express three types of asRNAs, which can regulate the Ty1 copy numbers in yeasts [29].

Recent studies including the FANTOM3 mouse transcriptome sequencing consortium identified natural antisense transcripts for more than 70% of transcription units (TUs), most of which represent non-protein-coding RNAs [30,31]. The existence and functional importance of asRNAs in various species have also been elucidated [31-34]. Various natural antisense RNAs (NATs) play important roles in the regulation of gene expression through diverse molecular mechanisms, such as X-chromosome inactivation (*Tsix*), genomic imprinting (*Air*), and trans-acting regulation (*HOTAIR* and *ANRIL*) of its sense strand expression [31,35-40]. Furthermore, abnormal expression of asRNAs is reported to be one of the risk factors in some diseases such as α-thalassemia, cardiac diseases, and Alzheimer’s disease [37,41,42]. Thus, the transcriptional control of asRNAs is considered to be a potential target for the development of new treatment strategies as well as the prevention of diseases.

Consequently, the identification and delineation of the precise primary structure and functions of HIV-1 asRNAs is urgently needed, because that information may provide new insights into the pathogenic mechanisms of HIV-1. In the present study, we identified an apparent major form of asRNAs, *ASP-L*, in HIV-1_NL4-3_ and HIV_IIIB_ infected cells. The results demonstrated that the *ASP-L* is localized in the nucleus and has a potential to negatively regulate HIV-1 replication. These findings suggest a novel mechanism that may play a role in the self-limiting replication of HIV-1.

## Results

### Mapping of potential antisense RNAs from HIV-1 proviral DNA

**Figure 1:**
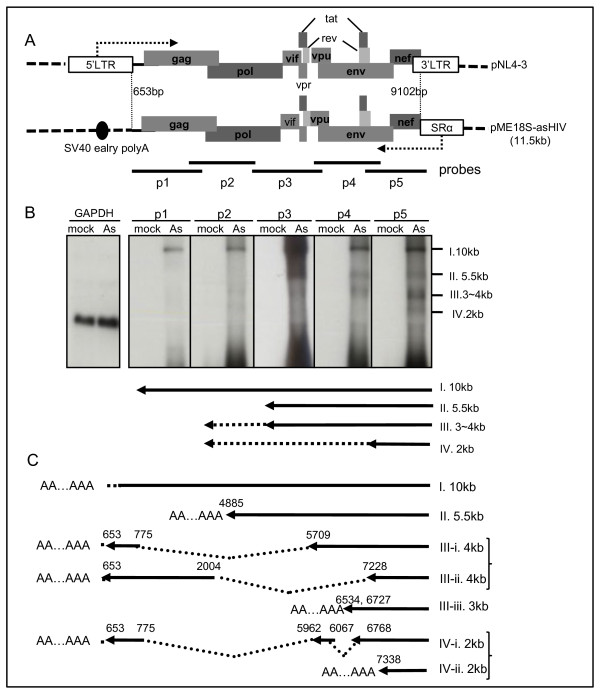
**Mapping of the potential antisense RNAs transcribed from the HIV-1 proviral DNA. (A)** A schematic diagram of the pME18S-asHIV plasmid. The antisense strand of HIV-1_NL4–3_ DNA was inserted downstream of the SRα promoter. **(B)** Results of Northern blot analysis for the detection of the RNAs from pME18S-asHIV. HEK293T cells were transfected with pME18S-asHIV or a mock vector, pME18S. Total RNA samples isolated from these cells were analyzed with region-specific probes described in A (p1–p5). *GAPDH* RNA was used as a loading control. The arrows described below are the positions of potential transcripts I–IV as described in the results. ‘Mock’ stands for the RNA sample extracted from cells transfected with a mock vector. ‘As’ stands for RNA samples extracted from cells transfected with pME18S-asHIV. **(C)** The summary of the transcription patterns in pME18S-asHIV-transfected cells. Spliced variants derived from antisense HIV-1 were identified by RT-PCR, and termination sites were determined by the 3′ RACE methods described in Additional file 1: Figure S1 and Additional file 2: Figure S2. Nucleotide numbering corresponds to the sense strand of HIV-1_NL4–3_-DNA.

Specific detection and identification of antisense transcripts of HIV-1 is difficult because of the presence of several sense transcripts [8,11]. Thus, we first attempted to characterize the candidate transcripts from the antisense strand of HIV DNA. We studied the transcripts in HEK293T cells that were transiently transfected with an expression plasmid, pME18S-asHIV, which contains the *gag* to *nef* region of HIV-1_NL4–3_ DNA in the antisense orientation was positioned between the RSV promoter and the SV40 poly (A) sites (Figure 1A). Total RNAs were extracted from the transfected cells and comprehensively analyzed by Northern blots using region specific probes against the HIV-1 p1–p5 regions (Figure 1B). Four major bands were detected that may represent potential antisense transcripts as shown in Figure 1B. We named these four bands I to IV based on their apparent molecular sizes. Transcript I is apparently a 10 kb genome-length transcript, and transcript II is a 5.5 kb transcript detected by p2–p5 probes. Transcript III appears to be a group of transcripts of 3–4 kb in size that hybridized with all probes except the p1 probe. Transcript IV is a 2 kb transcript that hybridized with p3, p4, and p5 probes, with a stronger signal to the p5 probe. Taken together, the results indicate that various transcripts can be transcribed from the antisense strand of HIV-1 DNA. Among these transcripts, those grouped in transcript III are expressed at high levels.

To characterize the detailed structure of these transcripts, we next performed RT-PCR analyses and 3′ RACE PCRs, and determined the spliced sites and the transcription termination sites (Additional file 1: Figure S1 and Additional file 2: Figure S2). Nucleotide sequence analyses of the amplified products revealed three kinds of spliced transcripts and four polyadenylation sites, suggesting seven kinds of potential asRNAs (Figure 1C). They are described as follow: Transcript I, a transcript with genome-length; Transcript II, a 5.5 kb transcript covering the nucleotide sequence from 9102 to 4885 of HIV-1_NL4–3_-sense DNA [GenBank: M19921.2] which corresponds to *ASP* mRNA reported by Landry *et al.*[11]; Transcripts III-i and III-ii, 4 kb transcripts terminating at the SV40 poly(A) sites in the vector; Transcript III-iii, a 3 kb transcript ranging from the *nef* to *env* regions of the sense strand (See Additional file 1: Figure S1. Determination of transcript III-iii); and Transcript IV, two transcripts terminating at the SV40 poly (A) sites (transcript IV-i) or at 7338 bp (transcript IV-ii) which corresponds to *ASP* mRNA reported by Michael *et al.*[8].

### Detection of HIV-1 antisense RNAs in infected cells

**Figure 2:**
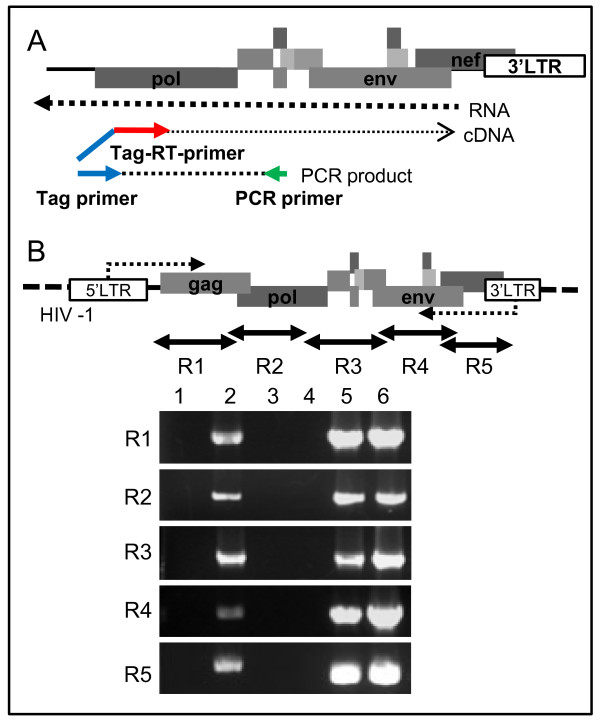
**Antisense-specific RT-PCR of HIV-1 RNA**. **(A)** A diagram describing the strand-specific RT-PCR method for HIV-1 asRNA. RNA samples were reverse-transcribed by an antisense-specific primer with a 5′ tag sequence (Tag-RT-primer). The synthesized cDNA samples were then amplified by PCR with a primer corresponding to the tag sequence (Tag primer) and a primer complementary to the target cDNA (PCR primer). **(B)** Detection of asRNAs in MAGIC-5A cells infected with HIV-1_NL4-3_. The top panel describes the position of R1–R5 region. Lower panels show the results of agarose gel electrophoresis of products of the antisense-specific RT-PCR targeting R1–R5 regions. PCR products were detected corresponding to all 5 regions studied. Lane 1, MAGIC-5A without virus; Lane 2, MAGIC-5A with HIV-1_NL4-3_; Lane 3, no RTase control; Lane 4, no RT primer control (a control for endogenous priming); Lane 5, PCR products with conventional primer pairs with cDNA samples synthesized by random primers (for testing sense and antisense RNA expressions); Lane 6, positive control (amplified from pNL4-3 plasmid DNA).

Based on the above results, we next examined whether the putative asRNAs are expressed in HIV-1-infected cells. Previous reports have documented that endogenous priming in the step of cDNA synthesis prevents strand-specific RT-PCR for analysis of intragenic asRNAs [11,43]. To eliminate the nonspecific amplification products, we utilized the strand-specific RT-PCR method (Figure 2A) [11,23], using MAGIC-5A cells infected with HIV-1 [44]. Antisense-specific RT-PCR was performed on five regions, R1–R5. Viral asRNAs were detected in all regions analyzed in MAGIC-5A infected with HIV-1_NL4–3_ (Figure 2B). However, no cDNA was amplified that corresponds to a spliced form of asRNA, including transcripts III-i, III-ii, and IV-i (Additional file 2: Figure S2). These results indicated that the antisense transcripts cover the region between 657 bp and 9094 bp and are unspliced forms in HIV-1 infected cells.

### Identification of a novel variant of *ASP* RNA, *ASP-L*

**Figure 3:**
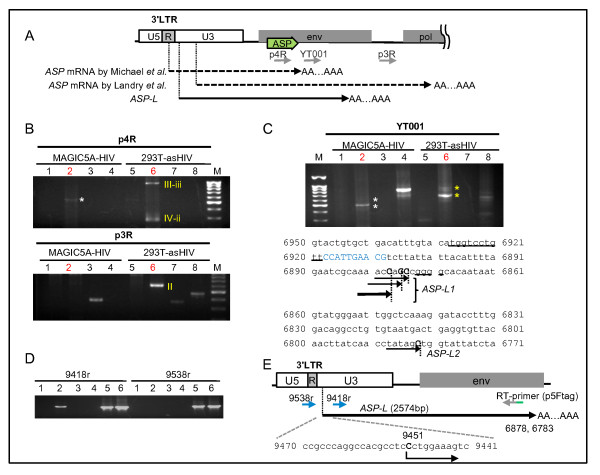
**Identification of an antisense RNA from HIV-1-infected cells. (A)** A schematic representation of the HIV-1 asRNAs and primers for 3' RACE. The black arrow represents the *ASP-L* transcript characterized in this study, and dotted arrows are the *ASP* mRNAs as previously described [8,14]*.* The bold green arrow indicates the position of the potential ORF, ASP. **(B and C)** Results of 3' RACE. ‘MAGIC5A-HIV’ stands for HIV-1 infected MAGIC-5A and ‘293 T-asHIV’ for HEK293T cells transfected with pME18S-asHIV. Lane 1 and 5, no infection control or mock transfected cells; Lane 2 and 6, cells with HIV-1 infection or transfected with pME18S-asHIV; Lane 3 and 7, no RTase control; Lane 4 and 8, no RT-primer control; M, 100 bp marker. **(B)** The result of 3′ RACE PCR using the p4R primer (upper panel) and the p3R primer (lower panel). The asterisk indicates the PCR product representing *ASP-L*. ‘II’, ‘III-iii’ and ‘IV-ii’ represent the PCR product derived from transcripts II, III-iii and IV-ii, respectively, described in Figure 1. **(C)** Upper panel: Results of semi-nested PCR with the YT001 primer. The white asterisks represent *ASP-L*-variant forms; the upper asterisk indicates *ASP-L2*, the lower one *ASP-L1*. The yellow asterisks represent transcript III-iii. Results of the sequencing analyses are shown in the bottom panel, where arrows and upper case letters indicate the termination sites. The bold arrow, end of the major transcript; the blue upper letters, a putative polyadenylation signal predicted by HCpolya (see Methods); the underline, putative polyadenylation elements of *ASP-L*. (D and E) Identification of transcription start site (TSS) of *ASP-L*. **(D)** Results of antisense-specific RT-PCR with two kinds of reverse primers. Locations of p5Ftag (Tag-RT-primer), 9418r, and 9538r are described in E. Lanes 1–6 are the same as in Figure 2B. **(E)** A schematic description of *ASP-L* RNA. The upper case letter C in the sequence indicates the position of *ASP-L* TSS.

The above results suggested that asRNAs of HIV-1, including transcripts I, II, III-iii and IV-ii, can be expressed in HIV-1-infected cells. To confirm the existence of these potential asRNAs and determine their primary structures, we next studied HIV-1 asRNAs in HIV-1-infected MAGIC-5A by 3′ RACE PCR analyses. Using the primer p4R that is located in the ASP open reading frame (ORF), a PCR product with an apparent molecular size of 700 bp was amplified in the infected MAGIC-5A cells (Figure 3A and B, lane 2 in the upper panel). In contrast to these results, no PCR products were amplified from the infected MAGIC-5A cells with the p3R primer (Figure 3B, lane 2 in the lower panel).

Semi-nested PCR using the PCR product by 3′ RACE with p4R primer produced one major product and one minor product, about 500 and 600 bp in size, in the infected MAGIC-5A cells. Analysis of the nucleotide sequences of these PCR products revealed a major poly (A) addition site that is located at the nucleotide position 6878 and other minor ones. Among the minor ones, one that extends to the nucleotide position 6783 corresponds to the larger PCR product. Thus, the results collectively showed that two groups of HIV-1 asRNAs were polyadenylated at nucleotide position 6878 (the major one) and 6783 (the minor one) in the *env* region (Figure 3C), which corresponds to the region of transcript III-iii (Figure 1C). *In silico* prediction identified a polyadenylation signal at nucleotide positions 6909 to 6918, which seems to be involved in the termination of the asRNA (Figure 3C).

Next, we studied the initiation site of the asRNA by using two reverse primers (9418r and 9538r). Antisense-specific RT-PCR with the primer 9418r successfully amplified a cDNA, whereas that with 9538r primer did not (Figure 3D). These results suggested that the transcriptional start site (TSS) is located between 9418 and 9538 of the proviral DNA. We next performed 5′ RACE to determine the TSS of the antisense transcript in the infected MAGIC-5A. The results indicated that the main TSS is at 9451 (Figure 3E), a finding supported by the successful amplification of cDNA by antisense-specific RT-PCR targeting the region between nucleotide positions of 6878 and 9451 (data not shown). *In silico* analysis predicted that this asRNA contains a few ORFs, of the previously reported *ASP* mRNA [8] and an extended 3′ UTR of approximately 500 bases compared with that of *ASP* mRNA (Figure 3A). The RT-PCR results suggested that this asRNA was mainly detected in infected MAGIC-5A cells (See Additional file 3: Figure S3).

Taken together, we have identified two new forms of asRNAs that are transcribed from the nucleotide position 9451 in the 3′ LTR U3 region of HIV DNA and terminated at nucleotide position 6878 or 6783 in the *env* region. We named these variants “*ASP* RNA-Long variant” (*ASP-L*)[GenBank: JQ866626]; a major variant terminated at 6878 is named as “*ASP-L1*,” and a minor variant terminated at 6783 as “*ASP-L2*.”

### Transcriptional activity of the LTR in the antisense orientation

**Figure 4:**
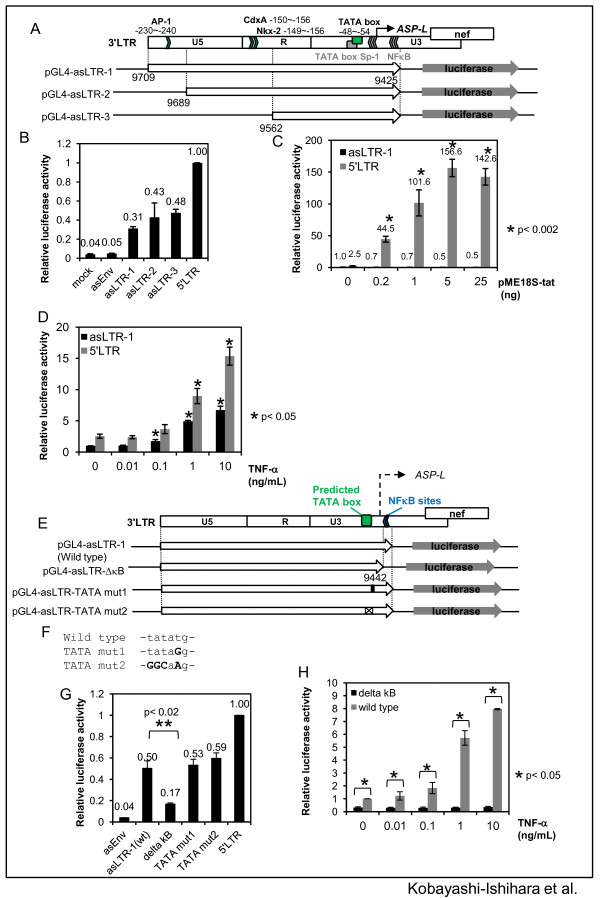
**Transcriptional activity of the HIV-1 3' LTR in the reverse orientation. (A)** A map of HIV-1 3' LTR and reporter plasmids. Top panel shows results of promoter prediction of the 5'-flanking region of the *ASP-L* TSS by TFsearch and Genetyx. Green box indicates putative TATA box. The sequence of the putative TATA box is described in F. The bottom panels are the structures of pGL4-asLTR-1, pGL4-asLTR-2 and pGL4-asLTR-3 plasmid. **(B)** Promoter activities of the LTR in the antisense orientation (asLTR). pGL4-5' LTR and pGL4-asEnv plasmids were used as positive and negative controls, respectively. mock, pGL-4.10; asEnv, pGL4-asEnv; asLTRs, pGL4-asLTR-1, pGL4-asLTR-2 and pGL4-asLTR-3; 5′ LTR, pGL4-5' LTR. The luciferase activities relative to that of pGL4-5' LTR are shown. **(C)** Effects of Tat protein on the asLTR promoter activity. A Tat expression plasmid, pME18S-tat, was co-transfected with pGL4-asLTR-1 or pGL4-5' LTR. **(D)** Dose–response effects of TNF-α on the asLTR promoter activity. At 12 h post-transfection of the reporters, cells were treated with various amounts of TNF-α (0–10 ng/mL) for 12 h. (**E-H**) Investigation of transcriptional regulatory elements in the asLTR. **(E)** A schematic description of series of asLTR mutant reporters. asLTR-ΔκB lacks two NF-κB binding sites. asLTR-TATA mut1 and mut2 contain mutated TATA box. **(F)** Sequences of the potential TATA box mutants. **(G)** Promoter activities of the asLTR mutants. The luciferase activities relative to that of pGL4-5' LTR are shown. **(H)** Effects of TNF-α on the ΔκΒ mutant. The experimental condition is identical to D. ‘delta kB’ stands for pGL4-sLTR-ΔκΒ. ‘wild type’ stands for pGL4-asLTR-1. The mean ± S.D. of quadruplicate (B) or triplicate (C, D, G, and H) experiments are shown. The asterisks shown in C, D, G, and H indicate statistical significance.

Since the transcription start sites (TSSs) of the newly characterized HIV-1 asRNAs are in the U3 region of 3′ LTR, the R-U5 region is expected to have antisense promoter activity. To study the promoter activity, we first performed a computational analysis of this region. The results revealed a potential TATA box at the nucleotide position −48 to −54 from the TSS as well as a couple of potential motifs that are recognized by transcription factors, such as CdxA, Nkx-2, and AP-1 (Figure 4A). To experimentally verify the promoter activity of this region, we performed luciferase reporter assays in Molt-4 cells using three kinds of constructs that have varying lengths of U3-R-U5 region (300 bp to140bp) in the antisense orientation (pGL4-asLTR-1, pGL4-asLTR-2, and pGL4-asLTR-3) (Figure 4A). Transfection of pGL4-asLTR-1 demonstrated a luciferase activity, the level of which was approximately one-third of that of the sense orientation LTR (pGL4-5′ LTR) (Figure 4B). Luciferase reporters having shorter regions (pGL4-asLTR-2 and pGL4-asLTR-3) also showed similar levels of promoter activities. On the other hand, a control construct that had a DNA fragment of the *env* region in the reverse orientation did not show any promoter activity.

To study the regulation of promoter activity of the LTR in the antisense orientation (asLTR), we next tested whether the viral accessory protein, Tat, or a cellular cytokine, TNF-α modulates the activity. Results of cotransfection experiments with a Tat expression plasmid did not show any activation of the antisense promoter, whereas the sense orientation LTR (pGL4-5′ LTR) responded to Tat in a dose-dependent manner, as expected (Figure 4C). On the other hand, TNF-α activated pGL4-asLTR-1 in a dose-dependent manner as was observed on the pGL4-5′ LTR (Figure 4D). TNF-α treatment of ACH-2 activated the expression of both strands (See Additional file 4: Figure S4).

The results shown in Figure 4D suggested the involvement of NF-κB in the regulation of 3′ LTR promoter activity in the antisense orientation. To examine this possibility, we prepared a mutant reporter (pGL4-asLTR-ΔκΒ, Figure 4E), which has a deletion of NF − κB binding motifs. The basal promoter activity of the asLTR-ΔκB was significantly decreased compared with that of wild type (Figure 4G) and lost responsiveness to TNF-α treatments (Figure 4H). We further tested the possible involvement of the putative TATA box using two kinds of mutant reporters, pGL4-asLTR-TATA mut1 and pGL4-asLTR-TATA mut2 (Figure 4E and F). pGL-4asLTR-TATA mut1 has a T to G point mutation at the potential TATA box, which lacks a TATA activity [45,46]. pGL-asLTR-TATA mut2 has mutations with a deletion of the TATA motif. The results demonstrated no difference in the basal promoter activity compared with that of wild type asLTR (Figure 4G).

### *ASP-L* expression in various types of cells infected with HIV-1

**Figure 5:**
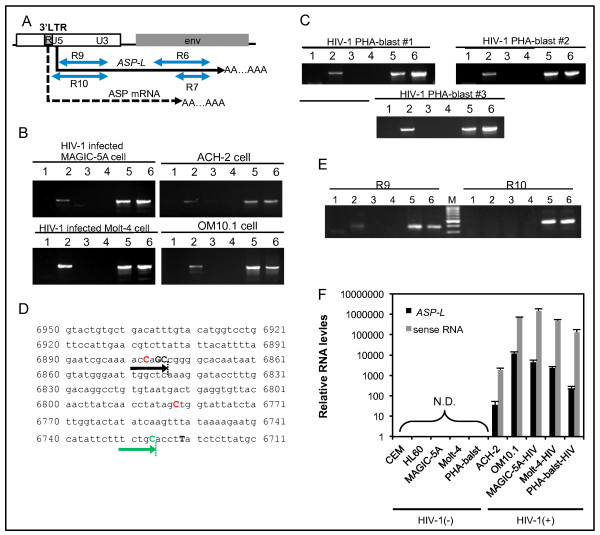
**Expression of the HIV-1 antisense RNA in various types of HIV-1-infected cells. (A)** A schematic representation of positions of *ASP-L* and *ASP* mRNA in the antisense strand of HIV-1_NL4-3_ genome. Arrows indicate the regions of the antisense-specific RT-PCR (R6, R7, R9, and R10). **(B and C)** Results of antisense-specific RT-PCR at R6 region. Total RNAs from HIV-1_NL4-3_-newly infected Molt-4 and MAGIC-5A cells, and ACH-2 and OM10.1 chronically infected cell lines were analyzed by the antisense-specific RT-PCR **(B)**. Results of HIV-1_NL4-3_ infected-PBMCs from three healthy individuals (HIV-1-PHA-blast #1–#3) are shown in **(C)**. Lane1, uninfected cell line or PBMC; Lane 2, HIV-1 infected cell line or PBMC; Lane 3, no RTase control; Lane 4, no RT primer control, Lane 5; amplified from cDNA synthesized with random primers; Lane 6, positive control (amplified from pNL4-3 plasmid DNA or cellular genomic DNA). **(D)** Termination sites of HIV_IIIB_*ASP-L*. Summary of 3' RACE analyses in OM10.1 and TPA-stimulated ACH-2 is shown. The upper letters mean transcription termination sites in black (ACH-2), green (OM10.1) and red (HIV-1_NL4-3_ infected MAGIC-5A cells). The black and green arrows are the major transcript in ACH-2 and OM10.1, respectively. **(E)** Determination of *ASP-L* TSS in HIV_IIIB_. The results of antisense-specific RT-PCR at R9 and R10 are shown. Lanes 1–6 are the same as in Figure 2B. (**F**) Relative expression levels of the antisense and sense transcripts. Results of the strand-specific qRT-PCR at R7 region are shown to measure the expression levels of the antisense and sense transcripts (sense RNA). Expression levels were normalized by the levels of *β-actin* gene expression. Results of triplicated experiments are shown with mean ± S.D.

To study the expression of HIV-1 asRNAs in various types of HIV-1-infected cells, we analyzed RNA samples from Molt-4 acutely infected with HIV-1_NL4–3_, as well as in ACH-2, and OM10.1 cell lines that are chronically infected with HIV-1_IIIB_[47,48] with the antisense-specific RT-PCR. In this experiment, we designed an antisense-specific RT-PCR with a Tag-RT-primer that does not amplify the *ASP* mRNA reported by Michael *et al.*[8] (Figure 5A). The asRNA expression was detected in all the cell lines examined (Figure 5B). Furthermore, we could also detect asRNA in the PHA-activated PBMCs infected with HIV-1_NL4–3_ (Figure 5C). 3′ RACE analyses revealed similar transcription termination in OM10.1 and activated ACH-2 cells, where HIV-1_IIB_ also had a conserved polyadenylation signal (Figure 5D). By antisense-specific RT-PCR analyses, we demonstrated that TSS of *ASP-L* from HIV-1_IIB_ was located between nucleotide positions 9441 and 9538 that correspond with that from HIV_NL4–3_ (Figure 5D). Collectively, *ASP-L* was demonstrated to be transcribed in cell lines with acute or chronic infection as well as in primary human PBMCs infected with HIV-1.

To evaluate the expression levels of the asRNA in various cells, quantitative analysis was performed using the strand-specific quantitative RT-PCR method (qRT-PCR) of the R7 region (Figure 5A and 5F). The results showed that the highest expression level was observed in OM10.1. The expression levels of HIV-1 asRNAs were shown to be 100–2,500 times less abundant than those in the sense RNA transcripts in all cells.

### Sub-cellular localization of HIV-1 antisense RNAs

**Figure 6:**
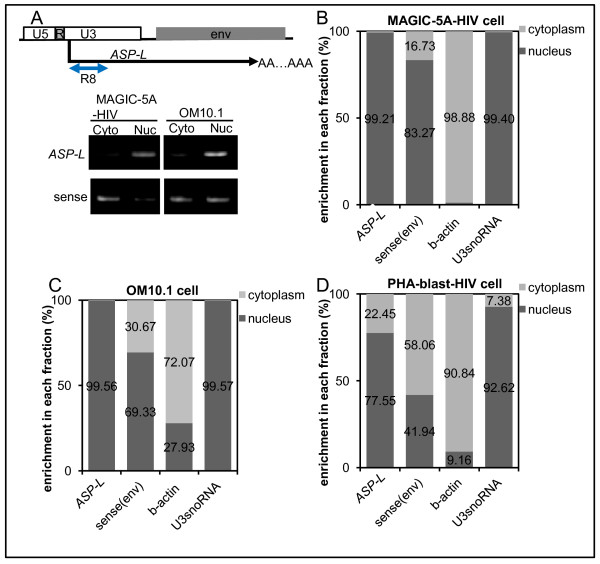
**Sub-cellular localization of HIV-1 antisense RNAs. (A)** Strand-specific RT-PCR of fractionated samples. An arrow in the upper panel indicates the region of PCR amplification (R8). Bottom panel, agarose gel electrophoresis of PCR products using samples of HIV-1-infected MAGIC-5A and chronically infected OM10.1 cells. Cyto, cytoplasmic RNA; Nuc, nuclear RNA; sense, results of sense strand-specific RT-PCR at R8. **(B–D)** Sub-cellular distribution of the asRNAs in various HIV-1-infected cells. Relative levels are presented in percentages for MAGIC-5A, OM10.1, and PHA-blast, as well as simultaneously measured controls. Results of *β-actin* cytoplasmic RNA and nuclear U3 snoRNA served as controls for fractionation efficiencies.

The subcellular localization of asRNAs could be a key to understand the possible functions of asRNAs [30,38,49]. We, therefore, studied the localization ratios of HIV-1 asRNAs between the cytoplasm and nucleus. For this purpose, HIV-1-infected MAGIC-5A and OM10.1 cells were fractionated into the cytoplasmic and nuclear portions, followed by RNA extraction. The results of antisense strand-specific RT-PCR at R8 region revealed that the majority of HIV-1 asRNAs was enriched in the nuclear fractions in both cell lines, whereas the sense strand transcripts did not show such a biased distribution (Figure 6A). To evaluate the distribution more quantitatively, we next employed qRT-PCR at the R7 region. The results revealed that more than 77% of the asRNA is located in the nuclei of various cells including primary PHA-activated PBMC (Figure 6B–D). This significantly biased nuclear localization was also confirmed in ACH-2 and HIV-1-infected Molt-4 (See Additional file 5: Figure S5. Sub-cellular localization of HIV-1 antisense RNAs in the HIV-1 infected T cell lines). Taken together, these results indicate that *ASP* RNAs are localized mainly in the nuclei of the acutely or chronically infected cell lines as well as in the nuclei of primary PBMCs newly infected by HIV-1.

### Inhibitory effects of the antisense RNA on HIV-1 replication

**Figure 7:**
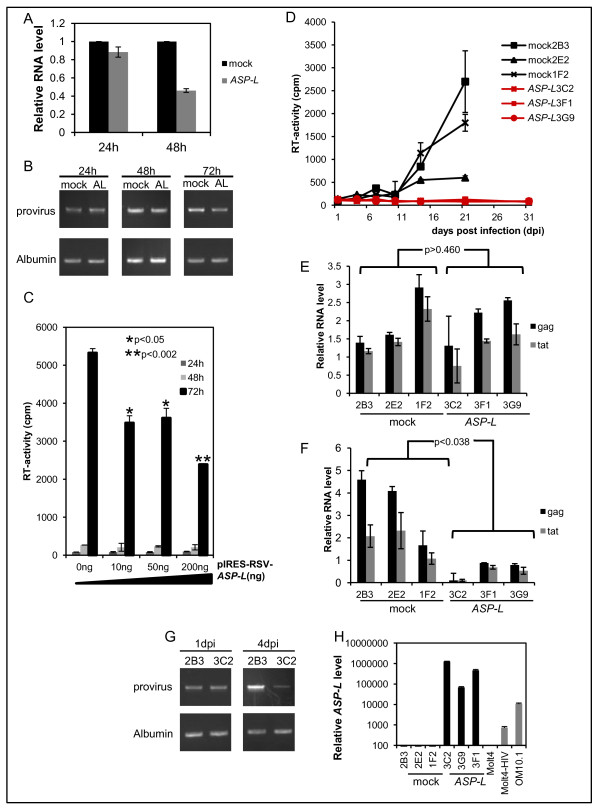
***ASP-L*-mediated inhibition of HIV-1 replication.** Results of transient transfection experiments are shown in A to C. **(A)** HIV-1 RNA levels at the indicated time points with prior transfection of the *ASP-L* expression vector, pIRES-RSV-*ASP-L*, or a vacant vector into MAGIC-5A cells. The relative expression levels of HIV-1 *gag* RNA are presented relative to that of HIV-1 *gag* RNA expressed in mock-transfected cells set as 1.0. **(B)** Agarose gel electrophoresis of PCR products of semi-quantitative PCR of genomic DNA samples harvested at the indicated time points. ‘AL’ stands for pIRES-RSV-*ASP-L* transfected cells. **(C)** The levels of viral particle production in culture supernatants evaluated by RT assays. MAGIC-5A cells were transfected with the indicated amounts of the *ASP-L* expression vector before infection with HIV-1. **(D–H)** Prolonged inhibition of HIV-1 replication in *ASP-L* expressing Molt-4 clones. Three clones that stably express *ASP-L* and three clones transfected with the vacant vector were used. **(D)** The levels of virus production evaluated by RT assays. (E and F) HIV-1 RNA levels at 1 day **(E)** and 4 days **(F)** post infection (dpi) measured by qRT-PCR. **(G)** Agarose gel electrophoresis of PCR products of semi-quantitative PCR of genomic DNA samples at 1 and 4 dpi.. **(H)** The relative expression levels of *ASP-L* that were measured by qRT-PCR.

To understand the function of HIV-1 asRNAs, we studied the effects of *ASP-L* on HIV-1 replication. MAGIC-5A cells were transiently transfected with an *ASP-L* expression vector, pIRES-RSV-*ASP-L*, or a vacant vector, followed by infection with HIV-1. At 48 h post-transfection (p.t.), the levels of *gag* RNA decreased in *ASP-L*-expressing cells compared to those in the control cells (Figure 7A). Semi-quantitative PCR analysis of the genomic DNA did not show any significant differences in the copy numbers of the proviral DNA at p.t. 24 h, whereas it showed decreased levels of proviral DNA in *ASP-L*-expressing cells at p.t. 48 and 72 h (Figure 7B). When the virus production was evaluated by RT assays with the culture supernatants, it was decreased in the *ASP-L*-expressing cells at p.t. 72 h with statistical significance (*p* < 0.002) (Figure 7C).

To study the inhibitory effects of *ASP-L* on HIV-1 replication in T-cells, we prepared three clones of Molt-4 that stably express *ASP-L* (3C2, 3F1, and 3G9). After infecting these cells with HIV-1, viral replication was evaluated by RT assays. The results demonstrated a significant repression of viral replication in the *ASP-L*-expressing cell lines for more than 30 days post HIV-1 infection (dpi) (Figure 7D). qRT-PCR analysis of the HIV-1 sense strand RNA did not show any significant differences in the levels of *gag* and *tat* RNAs at 1 dpi between the *ASP-L*-expressing cells and mock control cells; however, it demonstrated a 5-fold reductions in the levels of *gag* and *tat* RNAs at 4 dpi in the *ASP-L*-expressing cells compared to those levels in the control cells (Figure 7E and 7E). Semi-quantitative PCR of the genomic DNA did not show a significant difference in the proviral DNA levels between *ASP-L*-expressing and control cells at 1 dpi, whereas decreased levels of proviral DNA copies were shown in *ASP-L*-expressing cells at 4 dpi (Figure 7G). Among the stable *ASP-L*-expressing cell lines, the most significant inhibitory effect against HIV-1 replication was observed in clone 3C2 that expresses the highest levels of *ASP-L*, where the level of *ASP-L* was estimated to be about 120 times more abundant than that in OM10.1 (Figure 7F and H). Furthermore, the nuclear localization of *ASP-L* in the *ASP-L*-expressing clones was confirmed as described above (See Additional file 6: Figure S6. Sub-cellular localization of *ASP-L* in Molt-4 stably expressing *ASP-L*). Contrary to above results, the cells expressing the 3′ region of *ASP-L* showed no inhibitory effect on HIV-1 replication (See Additional file 7: Figure S7A-C).

### Upregulation of HIV-1 expression by knockdown of the endogenous antisense RNA

**Figure 8:**
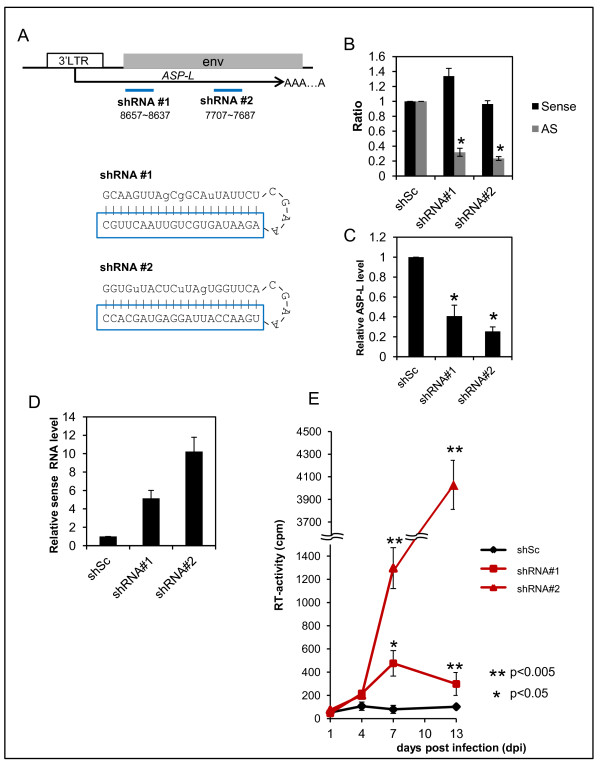
**Effects of endogenous HIV-1 antisense RNA on HIV-1 expression. (A)** Targeted regions of shRNA#1 and #2. Lower panel depicts secondary structures of expressed shRNAs predicted by m-fold. Lower case letters indicate mutated sites. Boxed sequences are guide strand for HIV-1 asRNAs. **(B)** Reporter-based confirmation of strand-specific knockdown. Luciferase reporter plasmids that contain sense HIV-1 sequence or antisense HIV-1 sequence were transfected into the shRNA-expressing Molt-4 cells. Relative changes of luciferase activities were calculated by pMIR-sense or AS *ASP-L*/pMIR-report. The mean ± S.D. of triplicate experiments are shown. Asterisks indicate statistical significance (*p* < 0.01). **(C)** Levels of endogenous HIV-1 asRNAs in HIV-1-infected shRNA expressing cells at 24 h of infection evaluated by strand-specific qRT-PCR at R7. Asterisks indicate statistical significance (*p* < 0.009). **(D)** HIV-1 sense RNA levels at 4 dpi measured by strand-specific qRT-PCR at R7 (*env* region). **(E)** Levels of viral particle production. At the indicated time points, cultured supernatant were collected and subjected to RT-assay. Asterisks indicate statistical significance.

As described above, the enforced expression of *ASP-L* downregulated the viral gene expression and replication. We then examined the biologic effects of *ASP-L* in HIV-1 infected cells. We established Molt-4 transformants that stably express short-hairpin RNAs (shRNAs) targeted to the HIV-1 asRNAs (shRNA#1 and shRNA#2, Figure 8A). To exclude the possible interference against sense strand RNAs, several mutated nucleotides were introduced into the passenger strands, which target to HIV-1 sense RNAs. First, we tested the specificity of these shRNAs using luciferase reporters having a sense or antisense sequence in these transformants. The two shRNAs specifically reduced the levels of luciferase activities of the reporters having *ASP-L* sequence, whereas the effects were not significant for the reporters having sense strand RNA (Figure 8B). Next, we examined the effects of these shRNAs in HIV-1 replication. The expression levels of the HIV-1 asRNAs in infected cells were suppressed in these cells compared with control cells that express a scrambled sequence (Figure 8C). qRT-PCR of RNA samples from the infected cells demonstrated significant enhancements of the levels of sense HIV-1 RNAs in the *ASP-L*-knockdown cells compared with that in the control cells (Figure 8D). Virus particle productions were evaluated by RT-assays using the supernatants of the infected cells. The RT activities were significantly enhanced in the samples of stable transformants expressing shRNA#1 and shRNA#2 compared with that of the control cells (Figure 8E).

## Discussion

In the present study, to clarify the natural structure of HIV-1 asRNAs, we employed a strategy that combines an artificial overexpression of antisense strand of HIV-1 and characterization of antisense transcripts in infected cells. The results revealed a natural form of asRNAs of HIV-1_NL4–3_ and HIV-1_IIIB_, *ASP-L*.

*ASP-L* appears to be a variant of previously reported *ASP* mRNA [8], in that it shares most of the region of the *ASP* mRNA, but lacks about 120 to 157 bases in the 5′ region and extends to the 3′ end by about 499 to 574 bases (Figure 3A). Previously, two groups reported structural analyses of *ASP* mRNAs (Figure 3A); first, Michael *et al.* isolated a single cDNA for *ASP* mRNA from a cDNA library prepared from A3.01 cells infected with HIV-1_IIIB_[8]. The transcript started at the nucleotide position 9608 and polyadenylated at the nucleotide position 7367 of the HXB2 strain, just after the TGA codon. Although a similar transcript was identified in our overexpression experiments (transcript IV-ii), this transcript was not identified in our experiments using HIV-1 infected cells (Figure 3B).

Secondly, Landry *et al.* reported the structure of another “*ASP* mRNA” in 293 T cells transfected with a 5′ LTR-deleted pNL4-3 [11]. The transcript started at various positions in the 5′ region of the 3′ LTR, and terminated in the *pol* region where they found a poly (A) signal at the nucleotide position 4908. This transcript appears to correspond to the transcript II in our overexpression experiments (Figure 1C); however, this transcript was detected only in the 293 T cells transfected by the antisense HIV-1 expression vector, but not detected in the HIV-1 infected cells in our experiments (Figure 3B), suggesting that this form of transcripts may be an artifact in overexpression experiments. As for transcript I, the asRNAs could not be detected by 3′ RACE method; nevertheless, its expression was suggested by the antisense-specific RT-PCR in the infected MAGIC-5A (Figure 2B). These results suggested that the expression level of transcript I is lower than that of *ASP-L*.

The results of reporter assays and 5′ RACE strongly support the notion that asRNAs of HIV-1 are transcribed from 3′ LTR sequence (Figures 3 and 4). Furthermore, consistent with previous reports [8,15,50], our reporter gene assays suggested that the asRNAs of HIV-1 could be transcribed from 3′ LTR in a TATA − independent and NF-κΒ − dependent manner (Figure 4D-H). On the other hand, the absence of TAR sequences in the antisense transcript may explain the absence of response to Tat (Figure 4C). These results also imply a possibility that the 5′ LTR may possess a promoter activity in the antisense direction, which might contribute to modulate the expression of flanking cellular genes [6]. In addition to our findings, there remains a possibility that the antisense promoter activity may also be influenced by flanking host sequences and the action of cellular transcription factors, since HIV-1 prefers to integrate into intergenic regions of actively transcribed genes [6,51]. Also, the transcription of asRNAs might be initiated within the host flanking sequences in some cases [20].

The results of antisense-specific qRT-PCR analyses indicated that the ratio of expression levels of HIV-1 asRNAs to those of the sense transcripts varied among the cells examined (1/100 to 1/2500, Figure 5F). Our result was similar to that of a previous study in which the authors estimated 0.9% abundance of HIV-1 asRNAs to the sense transcriptions [18]. The ratio of expressions was maintained at various stages of HIV-1 infection in Molt-4 (data not shown), implying a biological meaning of *ASP-L* in a life cycle of HIV-1. Taking our data described in Figures 7 and 8 into consideration, HIV-1 might retain a balanced expression of sense and antisense genes to avoid acute toxicity. In addition, the relative expression levels of HIV-1 asRNAs compared with those of *β-actin* were confirmed to be comparable to those of mRNAs of well-known protein encoding genes with important functions such as *Bcl-2Cyclin D1* and *IL-2* (data not shown).

To address the biological roles of the asRNA, we performed two experimental studies; first, using cells that stably overexpress *ASP-L*, we showed that *ASP-L* inhibits HIV-1 gene expression for a prolonged period (Figure 7). Since *ASP-L* expression did not affect the levels of HIV-1 DNA and RNA at 24 h post-transfection (Figure 7A, B, E, and G), *ASP-L* does not appear to inhibit the early processes of infection, such as viral entry and integration into the genomic DNA of target cells.

Next, we performed knockdown assays against the HIV asRNAs (Figure 8). The results suggested that asRNAs of HIV-1 including *ASP-L* might be involved in suppressing sense strand viral expression. Differences in the efficiency of viral replication between shRNA#1 and #2 may partly be attributed to their knockdown abilities (Figure 8B and C), although precise mechanisms need to be further studied. Taken collectively, the results suggest that the asRNAs may be a natural repressor for HIV-1 gene expression, which may contribute to a self-limited replication.

We demonstrated nuclear localization of *ASP-L* in the present study (Figure 6, Additional file 5: Figure S5 and Additional file 6: Figure S6). Furthermore, our data shown in Additional file 7: Figure S7 and Additional file 8 suggest that ASP protein may not be required for the antiviral function on HIV-1. These observations suggest a function of *ASP-L* that is exerted as a functional RNA. One previous study also raised a possibility that *ASP* mRNA may act as a functional RNA [13]. Recent reports demonstrated that the nuclear mRNA-like noncoding RNAs such as *Xist* and *HOTAIR* have important roles in regulating the sense strand gene expressions [39,52]. In addition, there is a possibility that *ASP-L* could be processed into small interference RNAs reducing HIV-1 replication [20].

However, considering a previous report that suggested the presence of antibodies that recognizes ASP protein in the sera of HIV-1 carriers [9], there remains a possibility that HIV asRNA may exert its functions both as a functional RNA and through protein(s) encoded by it. Considering the function of asRNA of human retroviruses, one intriguing example would be HBZ of HTLV-1. It has been reported that bZIP protein encoded by *HBZ* RNA can suppress transcription of HTLV-1 sense RNA [22,26], although antibodies that recognize HBZ have not been reported in sera of HTLV-1-infected individuals. In addition, some reports have suggested that *HBZ* RNA itself can regulate host cellular proliferation [24,25]. Further studies are required to elucidate detailed functional mechanisms of *ASP-L* and its putative translation product(s).

## Conclusions

We have identified a 2.6 kb asRNA of HIV-1, a variant of *ASP* mRNAs, which is transcribed from the U3 region of antisense strand of 3′ LTR and terminates in the *env* region. The asRNA was expressed in acutely or chronically infected cells and localized in the nuclei. The expression of the asRNA led to a prolonged inhibition of HIV-1 replication, and the knockdown of the *ASP-L* RNA significantly enhanced viral replication, suggesting that HIV-1 asRNA may be a novel factor for the self-limiting replication of HIV-1. Our finding of a new regulatory asRNA of HIV-1 will improve our understanding of regulatory mechanisms of viral replication, potentially providing a new approaches for anti-viral therapies.

## Availability of supporting data

The data sets supporting the results of this article are included within the article and its additional files.

## Methods

### Cells and viruses

HEK293T and MAGIC-5A cells [44] were maintained in DMEM (Dulbecco’s modified Eagle’s medium, Nissui) supplemented with 10% of heat-inactivated fetal bovine serum (FBS, GIBCO) and antibiotics. The following cell lines were maintained with RPMI 1640 medium with 10% FBS and antibiotics: Molt-4, CEM, HL60, ACH-2 (CEM cell-derived HIV-1_IIIB_ chronically infected cell line) [47] and OM10.1 (HL-60 cell-derived HIV-1_IIIB_ chronically infected cell line) [48]. Human PBMCs were isolated from whole blood of healthy donors by Ficoll-Paque gradient centrifugation (Amersham Biosciences) and stimulated with 10 ng/mL of PHA-P (Sigma) for 48 h. The activated PBMCs (PHA-blasts) were cultured in RPMI 1640 medium supplemented with 10% FBS, antibiotics and 20 U/mL of human recombinant IL-2 (R&D systems). HIV-1 NL4-3 strain was used for the infection studies. Viral particles were produced by calcium phosphate transfection of pNL4-3 plasmid in HEK293T cells as previously described [53].

### Expression vectors

Primers used for generating expression vectors are described in Additional file 9: Table S1. Primers used for this study. pME18S-asHIV was used for expression of the antisense strand of HIV-1_NL4–3_ (nucleotide position is 653 to 9102). The antisense strand of HIV-1 was obtained by PCR method with following primers: hiv-pnl-653 and hiv-pnl-9102, which are prepared based on the nucleotide sequence of pNL4-3 [53]. The PCR product was cloned into pGEM-Teasy (Promega) by TA method and sub-cloned into XbaI/NotI sites of pME18S [54].

The reporter gene plasmids, pGL4-asLTR-1, pGL4-asLTR-2, pGL4-asLTR-3 and pGL4-asLTR-∆κB were generated by inserting PCR amplified fragments with varying length of the upstream sequence of *ASP-L* TSS into SacI (blunted)/XhoI sites of pGL4.10 (Promega). The fragments correspond to the following nucleotide positions of HIV-1_NL4–3_: pGL4-asLTR-1, nucleotide position 9425 to 9709; pGL4-asLTR-2, 9425 to 9689; pGL4-asLTR-3, 9425 to 9562; pGL4-asLTR-∆κB, 9442 to 9709. pGL4-asEnv was generated by insertion of 200 bp length antisense fragment of *env* region that was amplified by p5R and 8514f primers, followed by XbaI/XhoI digestion. pGL4–5′ LTR vector for evaluating the transcriptional activity of the sense strand LTR was described previously [55]. The TATA box mutants, pGL4-asLTR-TATA mut1 and pGL4-asLTR-TATA mut2, were prepared by site-directed gene mutagenesis method [56,57] with primers described in additional file 9. These vectors were linearized by digestion with PstI or BstXI prior to transfection. pME18S-tat was used for Tat expression [58,59].

To investigate the effect of *ASP-L* on HIV-1 replication, we prepared an *ASP-L* expression vector, pIRES-RSV-*ASP-L*, using an expression vector pIRES-RSV that was derived from pIRESpuro3 (Clontech) containing RSV promoter. pIRES-RSV-*ASP-L* was generated by inserting a proviral DNA fragment that corresponds to the full-length of *ASP-L* at EcoRI/NotI sites. The *ASP-L* fragment was obtained by PCR from pNL4-3 with primers 6878f-NotI and 9460r.

### Transfections and HIV-1 infections

HEK293T cells (5 × 10^6^) were transfected with 4 μg of plasmid DNA, pME18S-asHIV or pME18S, as a control, by Lipofectamine reagent (Invitrogen) according to the protocol of the manufacturers. After 4 h incubation, the culture medium was changed and incubated additionally for 44 h. To obtain the RNA from HIV-1-infected cells, 1.5 × 10^6^ of MAGIC-5A cells were inoculated with HIV_NL4–3_ (3 × 10^3^ TCID_50_/50 ml) for 3 days. Total RNA was isolated by ISOGEN reagent (WAKO, Japan), followed by poly (A)^+^ RNA selection by oligo (dT) latex (Dai-ichi Kagaku Yakuhin, Japan).

For expression of *ASP-L* gene, 200 ng of pIRES-RSV or pIRES-RSV-*ASP-L* were transfected by Lipofectamine 2000 reagent (Invitrogen). After 4 h incubation, culture medium was changed, followed by inoculation of HIV_NL4–3_ at 200 TCID_50_/50 ml. After incubation for 18 h with HIV-1, cells were washed with DMEM to remove free viruses.

For establishment of T cell lines that stably express *ASP-L*, 5 × 10^6^ of Molt-4 cells were transfected with pIRES-RSV-*ASP-L* by electroporation, and several clones were selected by 0.5 μg/mL of Puromycin (Sigma). Among the Puromycin-resistant clones, three clones were selected based on the *ASP-L* expression confirmed by RT-PCR (3C2, 3F1, and 3G9). These clones were expanded and inoculated with HIV_NL4–3_ at MOI = 0.1. After 24 h of viral attachment, cells were washed by PBS and cultured in a 6-well plate.

### Northern blot analysis

Ten micrograms of total RNA samples were separated by 1% agarose-formaldehyde gel electrophoresis, and transferred onto a Biodin-A membrane (Pall). Hybridization was carried out with 7% SDS, 0.2 M Na_2_HPO_4_, and 1% BSA and isotope-labeled DNA probes for overnight at 65°C, followed by washing with 0.5 × SSC and 0.1% SDS at 65°C. Region specific DNA probes for p1–p5 regions were generated with PCR (See additional file 9: Table S1. Primers used for this study). The DNA fragments were TA-cloned into pGEM-Teasy vector, and the inserted DNA was purified from SmaI/XbaI digestion of the plasmid. The probes were labeled with [α-^32^P] dCTP by BcaBest labelling kit (TAKARA, Japan) according to the manufacture’s protocol.

### Strand-specific RT-PCR and quantitative RT-PCR

Primers for RT-PCR are described in the additional file 9. DNaseI-treated RNA samples were reverse-transcribed with Tag-RT-primer at 55°C for 50 min by SuperScript III reverse transcriptase (Invitrogen). Semi-quantitative RT-PCR was performed by AccuPrime DNA polymerase (Invitrogen) with the gene-specific primer and Tag primer (See also Figure 2A).

For strand-specific quantification, the cDNAs were analyzed by real-time PCR system (Thermal cycler Dice, TAKARA). The strand-specific quantitative PCR (qPCR) was performed by gene-specific primers and SYBRGreen (TAKARA). Standard curves for strand-specific qRT-PCR at R7 region were generated by linearized plasmids into which target strand-specific RT-PCR products were inserted. Levels of *b-actin* RNA were measured as internal controls [55].

### 3′ and 5′ RACE of antisense RNAs

Both 3′ and 5′ RACE methods were performed with 500 ng poly (A)^+^ RNA samples according to the manufacturer’s protocols (3′- and 5′-Full RACE Core Set, TAKARA). 1^st^ and 2^nd^ PCRs were performed by GeneTaq DNA polymerase (WAKO) with region-specific primers (See additional file 9: Table S1. primers used for this study).

### *In silico* analyses

Genetyx ver.10 and TFsearch were utilized for the promoter analysis of the HIV-1 asRNAs. For predicting ORFs and polyadenylation signals, the sequence of *ASP-L* was analysed by ORF Finder and HCpolya.

### Reporter gene assays

Linearized firefly reporter plasmid and the RSV-Renilla plasmid were co-transfected into 2 × 10^5^ of Molt-4 cells with by Lipofectamine2000 reagent. At 24 h post-transfection, cells were harvested and evaluated the promoter activities by measurement of luciferase activities (Dual-Luciferase Reporter Assay System, Promega). Representative results of quadruplicate or triplicate experiments are presented with the mean and S.D. Treatment of TNF-α (0–10 ng/ml) was performed at 12 h post-transfection and the cells were incubated for an additional 12 h.

### Sub-cellular fractionation

Cultured cells were washed with PBS and lysed with lysis buffer (10 mM Tris–HCl, pH7.5, 10 mM NaCl, 1.5 mM MgCl_2_, 10 mM Vanadyl Complex, 1% NP-40) on ice for 5 min. After centrifugation in 3,000 rpm for 5 min at 4 Cº, cytoplasmic supernatant and pelleted nuclei were separated and resuspended in ISOGEN-LS (WAKO) for RNA extraction. Relative antisense and sense strand RNA levels were measured by the strand-specific qRT-PCR method described above and calculated the enrichment of RNA levels in each compartment as below. Distribution of interested RNA was calculated as follows: (% of enrichment in each fraction) = (level of RNA in nuclear or cytoplasmic fraction)/(total levels of RNA in nuclear and cytoplasmic fractions) × 100. The efficiency of the fractionation procedure was confirmed by testing the distributions of *β-actin* cytoplasmic RNA and U3 small nucleolar RNA (U3 snoRNA) [49].

### Measurement of virus production

Viral replication was evaluated by measurements of free virions in the culture media with RT assay [60]. Levels of intracellular *gag* and *tat* RNAs were measured by qRT-PCR as described previously [55]. Proviral loads were measured by PCR with p1R and p2F primers (See additional file 9: Table S1. primers used for this study) from genomic DNA samples isolated by QIAamp DNA Blood Mini Kit (Qiagen). Albumin DNA levels were used as a loading control [55].

### Retroviral transduction and strand-specific RNA interference

Recombinant retroviruses carrying shRNA#1 and #2 were constructed by annealed double-strand oligonucleotides (shRNA#1, 5′-GCAAGTTAgCgGCAtTATTCTCGAAAGAATAGTGCTGTTAACTTGC-3′; shRNA#2, 5′-GGTGtTACTCtTAgTGGTTCACGAATGAACCATTAGGAGTAGCACC-3′) into a pSIN-sihU6 vector (TAKARA). The sequence of scrambled RNA and detailed procedure of retroviral production were as described previously [55]. After transduction of recombinant viruses and G418 selection, cells were expanded and inoculated with HIV_NL4–3_ at MOI = 0.1. After 24 hours of viral attachment, cells were washed with PBS and then cultured in a 12-well culture plate.

To confirm the strand-specific knockdown by shRNAs, the cells were transfected with pMIR-REPORT (empty plasmid, Ambion), pMIR-sense *ASP-L* or pMIR-AS *ASP-L*, respectively. These reporters include sense or antisense HIV-1 sequence in the 3′UTR of luciferase gene.

## Abbreviations

asRNA, antisense RNA; HIV-1, Human immunodeficiency virus type 1; HTLV-1, Human T-cell leukemia virus type 1; HBZ, HTLV-1 b-ZIP protein; LTR, Long terminal repeat; PBMCs, Peripheral Blood Mononuclear Cells; PBS, Phosphate-buffered saline; PHA-P, Phytohemagglutinin-P; Tat, Trans-activator of transcription; TNF-α, Tumor necrosis factor-α.

## Competing interests

The authors declare that they have no competing interests.

## Authors’ contributions

MKI improved the strand-specific RT-PCR and carried out most of the experiments and drafted the manuscript. MY advised the experimental designs and helped in drafting and finalizing the manuscripts. TH and YM helped isolate PBMC and extract cellular RNAs. RT advised the protocol used in this study and helped subcloning of shRNA-expression vectors. AM, KN, TY and TI advised the experimental design and protocols used in this study. TW conceived the study, designed the experiments and helped in drafting and finalizing the manuscripts. All authors read and approved the final manuscript.

## Supplementary Material

Additional file 1: **Figure S1. Determination of transcript III-iii. (A)** Results of 3' RACE. Top panel summarizes the results. Agarose gel electrophoresis of 3' RACE PCR products is shown in Figure 3B. The results of the sequence analyses are shown in the bottom panel. Bars and arrows indicate the identified termination sites. The bold arrow shows the major transcript. The upper case letters and arrows in the sequence indicate the termination sites of transcript III-iii. **(B)** Termination positions of transcript III-iii described in the context of pME18S-asHIV. Click here for file

Additional file 2: **Figure S2. Detection of spliced HIV-1 antisense RNAs. (A)** A map of primer pairs at R11 and R12. **(B-C)** Results of agarose gel electrophoresis of antisense-specific RT-PCR products at R11 (**B**) and R12 (**C**). Expected PCR products derived from spliced transcripts were approximately 2 kb (**B**) and 400 bp (**C**), respectively (indicated by asterisks in lane 2), which are shorter than that of full-length (6 kb at R11 and 5 kb at R12). Experiments were performed using total RNAs from HEK293T with pME-18 S-asHIV (293 T-asHIV) (left panel) and HIV-1-infected MAGIC-5A (right panel). Lane 1 and 7, cells transfected with a mock vector or no infection control; Lane 2 and 8, cells with pME18S-asHIV or HIV-1 infection; Lane 3 and 9, no RTase control; Lane 4 and 10, no RT primer control; Lane 5 and 11, PCR products with conventional primer pairs with cDNA samples synthesized by random primers; Lane 6 and 12, positive control (amplified from pME18S-asHIV plasmid DNA, or from pNL4-3 plasmid DNA); M, 100 bp marker; Λ,Λ/Hind III marker. Click here for file

Additional file 3: **Figure S3. HIV-1 antisense RNA pattern in infected cells.** MAGIC-5A cells were infected with HIV-1_NL4–3_ and then analyzed antisense RNAs by antisense-specific RT-PCR at regions R9 (for original *ASP* mRNA) and R10 (for *ASP-L*). Representative results (n = 4) were shown. *ASP-L* was mainly detected. Lanes 1–6 are the same as in Figure 2B. Click here for file

Additional file 4: **Figure S4. Transcriptional activation of HIV-1 antisense RNAs by TNF-α treatment.** ACH-2 was treated with TNF-α (10 ng/mL) for 24 h. Total RNAs were extracted and analyzed by strand-specific qRT-PCR at R7. The asterisks denote statistical significance relative to the untreated control (*p* < 0.02). Click here for file

Additional file 5: **Figure S5. Sub-cellular localization of HIV-1 antisense RNAs in the HIV-1 infected T cell lines.** Results of subcellular localization analysis of T-cell lines. RNA samples were prepared from cytoplasmic and nuclear fractions of ACH-2 cells and HIV-1_NL4–3_-infected Molt-4 cells as described in the text. Click here for file

Additional file 6: **Figure S6. Sub-cellular localization of*ASP-L*in the Molt-4 cells stably expressing*ASP-L.*** RNA samples were prepared from cytoplasmic and nuclear fractions of Clone 3G9 cells. Results of the quantitative measurement of RT-PCR at R7 are presented from cDNAs synthesized with random primers. Fractionation efficiencies were confirmed by measuring the levels of *β-actin* cytoplasmic RNA and nuclear U3 snoRNA. Click here for file

Additional file 7: **Figure S7. Inhibitory effects of full-length*ASP-L*RNA on HIV-1 replication. (A)***ASP-L* mutants. *ASP-L*3′ is a portion of *ASP-L* bearing the ASP-coding region. *ASP-L*_∆ATG_ contains an A to T mutation at the start codon of ASP. *ASP-L*_C-stop_ contains a C to A mutation at the seventh codon of ASP to convert Cysteine into a stop codon. Detailed sequences are provided in the right panels. Upper cases in the nucleotide sequences show the mutated sites. **(B–C)** Effects of ASP on HIV-1 replication. **(B)** Expression levels of *ASP-L*3′ measured by qRT-PCR at R7. mock, Clone 2B3 with the empty vector; wt, Clone 3C2 stably expressing wild type *ASP-L* (Figure 7D–G); c9F and c5D, established Molt-4 clones that stably express *ASP-L*3′; Molt4, uninfected Molt-4 cells; Molt4-HIV, HIV-1_NL4–3_ infected Molt-4. **(C)** HIV-1 RNA levels at 4 days post HIV-1_NL4–3_ infection. HIV-1 RNA levels were evaluated by qRT-PCR with *gag* and *tat* genes (mean ± S.D). **(D)** Effects of *ASP-L* RNA on HIV-1 replication. 50 ng or 200 ng of pIRES-RSV-*ASP-L*_∆ATG_ (dATG) or pIRES-RSV-*ASP-L*_C-stop_ (C-stop) was transfected into MAGIC-5A, followed by HIV-1_NL4–3_ infection. Viral production levels were evaluated by RT assays with the supernatants at 72 h post-transfection. **(E)** HIV-1 *gag* RNA levels at 48 h post-transfection measured by qRT-PCR.’mock’ stands for MAGIC-5A with empty vector. ‘wt’ stands for MAGIC-5A with pIRES-RSV-*ASP-L*. Click here for file

Additional file 8: **Supplemental materials and methods.** Supplemental materials and methods for expression vectors in Figure S7 are described. Click here for file

Additional file 9: Table S1. Primers used for this study. Click here for file

## References

[B1] Tasara T Hottiger MO Hubscher U Functional genomics in HIV-1 virus replication: protein-protein interactions as a basis for recruiting the host cell machinery for viral propagation Biol Chem 2001 382 7 993 999 11530943 10.1515/BC.2001.125

[B2] Trkola A HIV-host interactions: vital to the virus and key to its inhibition Curr Opin Microbiol 2004 7 4 407 411 10.1016/j.mib.2004.06.002 15358260

[B3] Fanales-Belasio E Raimondo M Suligoi B Butto S HIV virology and pathogenetic mechanisms of infection: a brief overview Ann Ist Super Sanita 2010 46 1 5 14 20348614 10.4415/ANN_10_01_02

[B4] Van Lint C Emiliani S Ott M Verdin E Transcriptional activation and chromatin remodeling of the HIV-1 promoter in response to histone acetylation EMBO J 1996 15 5 1112 1120 8605881 PMC450009

[B5] Bennasser Y Yeung ML Jeang KT HIV-1 TAR RNA subverts RNA interference in transfected cells through sequestration of TAR RNA-binding protein, TRBP J Biol Chem 2006 281 38 27674 27678 10.1074/jbc.C600072200 16887810

[B6] Colin L Van Lint C Molecular control of HIV-1 postintegration latency: implications for the development of new therapeutic strategies Retrovirology 2009 6 111 10.1186/1742-4690-6-111 19961595 PMC2797771

[B7] Miller RH Human immunodeficiency virus may encode a novel protein on the genomic DNA plus strand Science 1988 239 4846 1420 1422 10.1126/science.3347840 3347840

[B8] Michael NL Vahey MT d’Arcy L Ehrenberg PK Mosca JD Rappaport J Redfield RR Negative-strand RNA transcripts are produced in human immunodeficiency virus type 1-infected cells and patients by a novel promoter downregulated by Tat J Virol 1994 68 2 979 987 8289399 10.1128/jvi.68.2.979-987.1994 PMC236536

[B9] Vanhee-Brossollet C Thoreau H Serpente N D’Auriol L Levy JP Vaquero C A natural antisense RNA derived from the HIV-1 env gene encodes a protein which is recognized by circulating antibodies of HIV + individuals Virology 1995 206 1 196 202 10.1016/S0042-6822(95)80034-4 7831774

[B10] Ludwig LB Ambrus JL Krawczyk KA Sharma S Brooks S Hsiao CB Schwartz SA Human Immunodeficiency Virus-Type 1 LTR DNA contains an intrinsic gene producing antisense RNA and protein products Retrovirology 2006 3 80 10.1186/1742-4690-3-80 17090330 PMC1654176

[B11] Landry S Halin M Lefort S Audet B Vaquero C Mesnard JM Barbeau B Detection, characterization and regulation of antisense transcripts in HIV-1 Retrovirology 2007 4 71 10.1186/1742-4690-4-71 17910760 PMC2099442

[B12] Peeters A Lambert PF Deacon NJ A fourth Sp1 site in the human immunodeficiency virus type 1 long terminal repeat is essential for negative-sense transcription J Virol 1996 70 10 6665 6672 8794302 10.1128/jvi.70.10.6665-6672.1996 PMC190708

[B13] Tagieva NE Vaquero C Expression of naturally occurring antisense RNA inhibits human immunodeficiency virus type 1 heterologous strain replication J Gen Virol 1997 78 Pt 10 2503 2511 9349471 10.1099/0022-1317-78-10-2503

[B14] Briquet S Vaquero C Immunolocalization studies of an antisense protein in HIV-1-infected cells and viral particles Virology 2002 292 2 177 184 10.1006/viro.2001.1224 11878921

[B15] Bentley K Deacon N Sonza S Zeichner S Churchill M Mutational analysis of the HIV-1 LTR as a promoter of negative sense transcription Arch Virol 2004 149 12 2277 2294 10.1007/s00705-004-0386-8 15338321

[B16] Bansal A Carlson J Yan J Akinsiku OT Schaefer M Sabbaj S Bet A Levy DN Heath S Tang J Kaslow RA Walker BD Ndung’u T Goulder PJ Heckerman D Hunter E Goepfert PA CD8 T cell response and evolutionary pressure to HIV-1 cryptic epitopes derived from antisense transcription J Exp Med 2010 207 1 51 59 10.1084/jem.20092060 20065064 PMC2812545

[B17] Yeung ML Bennasser Y Watashi K Le SY Houzet L Jeang KT Pyrosequencing of small non-coding RNAs in HIV-1 infected cells: evidence for the processing of a viral-cellular double-stranded RNA hybrid Nucleic Acids Res 2009 37 19 6575 6586 10.1093/nar/gkp707 19729508 PMC2770672

[B18] Lefebvre G Desfarges S Uyttebroeck F Munoz M Beerenwinkel N Rougemont J Telenti A Ciuffi A Analysis of HIV-1 expression level and sense of transcription by high-throughput sequencing of the infected cell J Virol 2011 85 13 6205 6211 10.1128/JVI.00252-11 21507965 PMC3126515

[B19] Clerc I Laverdure S Torresilla C Landry S Borel S Vargas A Arpin-Andre C Gay B Briant L Gross A Barbeau B Mesnard JM Polarized expression of the membrane ASP protein derived from HIV-1 antisense transcription in T cells Retrovirology 2011 8 74 10.1186/1742-4690-8-74 21929758 PMC3182985

[B20] Schopman NC Willemsen M Liu YP Bradley T van Kampen A Baas F Berkhout B Haasnoot J Deep sequencing of virus-infected cells reveals HIV-encoded small RNAs Nucleic Acids Res 2012 40 1 414 427 10.1093/nar/gkr719 21911362 PMC3245934

[B21] Larocca D Chao LA Seto MH Brunck TK Human T-cell leukemia virus minus strand transcription in infected T-cells Biochem Biophys Res Commun 1989 163 2 1006 1013 10.1016/0006-291X(89)92322-X 2476979

[B22] Gaudray G Gachon F Basbous J Biard-Piechaczyk M Devaux C Mesnard JM The complementary strand of the human T-cell leukemia virus type 1 RNA genome encodes a bZIP transcription factor that down-regulates viral transcription J Virol 2002 76 24 12813 12822 10.1128/JVI.76.24.12813-12822.2002 12438606 PMC136662

[B23] Cavanagh MH Landry S Audet B Arpin-Andre C Hivin P Pare ME Thete J Wattel E Marriott SJ Mesnard JM Barbeau B HTLV-I antisense transcripts initiating in the 3'LTR are alternatively spliced and polyadenylated Retrovirology 2006 3 15 10.1186/1742-4690-3-15 16512901 PMC1459196

[B24] Satou Y Yasunaga J Yoshida M Matsuoka M HTLV-I basic leucine zipper factor gene mRNA supports proliferation of adult T cell leukemia cells Proc Natl Acad Sci U S A 2006 103 3 720 725 10.1073/pnas.0507631103 16407133 PMC1334651

[B25] Arnold J Zimmerman B Li M Lairmore MD Green PL Human T-cell leukemia virus type-1 antisense-encoded gene, Hbz, promotes T-lymphocyte proliferation Blood 2008 112 9 3788 3797 10.1182/blood-2008-04-154286 18689544 PMC2572803

[B26] Matsuoka M Green PL The HBZ gene, a key player in HTLV-1 pathogenesis Retrovirology 2009 6 71 10.1186/1742-4690-6-71 19650892 PMC2731725

[B27] Briquet S Richardson J Vanhee-Brossollet C Vaquero C Natural antisense transcripts are detected in different cell lines and tissues of cats infected with feline immunodeficiency virus Gene 2001 267 2 157 164 10.1016/S0378-1119(01)00404-8 11313142

[B28] Rasmussen MH Ballarin-Gonzalez B Liu J Lassen LB Fuchtbauer A Fuchtbauer EM Nielsen AL Pedersen FS Antisense transcription in gammaretroviruses as a mechanism of insertional activation of host genes J Virol 2010 84 8 3780 3788 10.1128/JVI.02088-09 20130045 PMC2849499

[B29] Matsuda E Garfinkel DJ Posttranslational interference of Ty1 retrotransposition by antisense RNAs Proc Natl Acad Sci U S A 2009 106 37 15657 15662 10.1073/pnas.0908305106 19721006 PMC2735561

[B30] Sasaki YT Ideue T Sano M Mituyama T Hirose T MENepsilon/beta noncoding RNAs are essential for structural integrity of nuclear paraspeckles Proc Natl Acad Sci U S A 2009 106 8 2525 2530 10.1073/pnas.0807899106 19188602 PMC2650297

[B31] Katayama S Tomaru Y Kasukawa T Waki K Nakanishi M Nakamura M Nishida H Yap CC Suzuki M Kawai J Suzuki H Carninci P Hayashizaki Y Wells C Frith M Ravasi T Pang KC Hallinan J Mattick J Hume DA Lipovich L Batalov S Engstrom PG Mizuno Y Faghihi MA Sandelin A Chalk AM Mottagui-Tabar S Liang Z Lenhard B Wahlestedt C RIKEN Genome Exploration Research Group, Genome Science Group (Genome Network Project Core Group), FANTOM Consortium Antisense transcription in the mammalian transcriptome Science 2005 309 5740 1564 1566 16141073 10.1126/science.1112009

[B32] Wagner EG Altuvia S Romby P Antisense RNAs in bacteria and their genetic elements Adv Genet 2002 46 361 398 11931231 10.1016/s0065-2660(02)46013-0

[B33] Georg J Honsel A Voss B Rennenberg H Hess WR A long antisense RNA in plant chloroplasts New Phytol 2010 186 3 615 622 10.1111/j.1469-8137.2010.03203.x 20202127

[B34] Unver T Bakar M Shearman RC Budak H Genome-wide profiling and analysis of Festuca arundinacea miRNAs and transcriptomes in response to foliar glyphosate application Mol Genet Genomics 2010 283 4 397 413 10.1007/s00438-010-0526-7 20213187

[B35] Lyle R Watanabe D te Vruchte D Lerchner W Smrzka OW Wutz A Schageman J Hahner L Davies C Barlow DP The imprinted antisense RNA at the Igf2r locus overlaps but does not imprint Mas1 Nat Genet 2000 25 1 19 21 10.1038/75546 10802648

[B36] Zhao J Sun BK Erwin JA Song JJ Lee JT Polycomb proteins targeted by a short repeat RNA to the mouse X chromosome Science 2008 322 5902 750 756 10.1126/science.1163045 18974356 PMC2748911

[B37] Faghihi MA Modarresi F Khalil AM Wood DE Sahagan BG Morgan TE Finch CE St Laurent G Kenny PJ Wahlestedt C Expression of a noncoding RNA is elevated in Alzheimer’s disease and drives rapid feed-forward regulation of beta-secretase Nat Med 2008 14 7 723 730 10.1038/nm1784 18587408 PMC2826895

[B38] Faghihi MA Wahlestedt C Regulatory roles of natural antisense transcripts Nat Rev Mol Cell Biol 2009 10 9 637 643 10.1038/nrm2738 19638999 PMC2850559

[B39] Gupta RA Shah N Wang KC Kim J Horlings HM Wong DJ Tsai MC Hung T Argani P Rinn JL Wang Y Brzoska P Kong B Li R West RB van de Vijver MJ Sukumar S Chang HY Long non-coding RNA HOTAIR reprograms chromatin state to promote cancer metastasis Nature 2010 464 7291 1071 1076 10.1038/nature08975 20393566 PMC3049919

[B40] Yap KL Li S Munoz-Cabello AM Raguz S Zeng L Mujtaba S Gil J Walsh MJ Zhou MM Molecular interplay of the noncoding RNA ANRIL and methylated histone H3 lysine 27 by polycomb CBX7 in transcriptional silencing of INK4a Mol Cell 2010 38 5 662 674 10.1016/j.molcel.2010.03.021 20541999 PMC2886305

[B41] Tufarelli C Stanley JA Garrick D Sharpe JA Ayyub H Wood WG Higgs DR Transcription of antisense RNA leading to gene silencing and methylation as a novel cause of human genetic disease Nat Genet 2003 34 2 157 165 10.1038/ng1157 12730694

[B42] Luther HP Role of endogenous antisense RNA in cardiac gene regulation J Mol Med 2005 83 1 26 32 10.1007/s00109-004-0613-5 15592803

[B43] Haddad F Qin AX Giger JM Guo H Baldwin KM Potential pitfalls in the accuracy of analysis of natural sense-antisense RNA pairs by reverse transcription-PCR BMC Biotechnol 2007 7 21 10.1186/1472-6750-7-21 17480233 PMC1876213

[B44] Hachiya A Aizawa-Matsuoka S Tanaka M Takahashi Y Ida S Gatanaga H Hirabayashi Y Kojima A Tatsumi M Oka S Rapid and simple phenotypic assay for drug susceptibility of human immunodeficiency virus type 1 using CCR5-expressing HeLa/CD4(+) cell clone 1–10 (MAGIC-5) Antimicrob Agents Chemother 2001 45 2 495 501 10.1128/AAC.45.2.495-501.2001 11158746 PMC90318

[B45] Bell SD Brinkman AB van der Oost J Jackson SP The archaeal TFIIEalpha homologue facilitates transcription initiation by enhancing TATA-box recognition EMBO Rep 2001 2 2 133 138 10.1093/embo-reports/kve021 11258705 PMC1083817

[B46] Bjornsdottir G Myers LC Minimal components of the RNA polymerase II transcription apparatus determine the consensus TATA box Nucleic Acids Res 2008 36 9 2906 2916 10.1093/nar/gkn130 18385157 PMC2396422

[B47] Folks TM Clouse KA Justement J Rabson A Duh E Kehrl JH Fauci AS Tumor necrosis factor alpha induces expression of human immunodeficiency virus in a chronically infected T-cell clone Proc Natl Acad Sci U S A 1989 86 7 2365 2368 10.1073/pnas.86.7.2365 2784570 PMC286913

[B48] Butera ST Perez VL Wu BY Nabel GJ Folks TM Oscillation of the human immunodeficiency virus surface receptor is regulated by the state of viral activation in a CD4+ cell model of chronic infection J Virol 1991 65 9 4645 4653 1678437 10.1128/jvi.65.9.4645-4653.1991 PMC248919

[B49] Berteaux N Aptel N Cathala G Genton C Coll J Daccache A Spruyt N Hondermarck H Dugimont T Curgy JJ Forne T Adriaenssens E A novel H19 antisense RNA overexpressed in breast cancer contributes to paternal IGF2 expression Mol Cell Biol 2008 28 22 6731 6745 10.1128/MCB.02103-07 18794369 PMC2573296

[B50] van der Sluis RM Pollakis G van Gerven ML Berkhout B Jeeninga RE Latency profiles of full length HIV-1 molecular clone variants with a subtype specific promoter Retrovirology 2011 8 73 10.1186/1742-4690-8-73 21923919 PMC3182984

[B51] Kilareski EM Shah S Nonnemacher MR Wigdahl B Regulation of HIV-1 transcription in cells of the monocyte-macrophage lineage Retrovirology 2009 6 118 10.1186/1742-4690-6-118 20030845 PMC2805609

[B52] Ohhata T Hoki Y Sasaki H Sado T Crucial role of antisense transcription across the Xist promoter in Tsix-mediated Xist chromatin modification Development 2008 135 2 227 235 18057104 10.1242/dev.008490

[B53] Adachi A Gendelman HE Koenig S Folks T Willey R Rabson A Martin MA Production of acquired immunodeficiency syndrome-associated retrovirus in human and nonhuman cells transfected with an infectious molecular clone J Virol 1986 59 2 284 291 3016298 10.1128/jvi.59.2.284-291.1986 PMC253077

[B54] Takebe Y Seiki M Fujisawa J Hoy P Yokota K Arai K Yoshida M Arai N SR alpha promoter: an efficient and versatile mammalian cDNA expression system composed of the simian virus 40 early promoter and the R-U5 segment of human T-cell leukemia virus type 1 long terminal repeat Mol Cell Biol 1988 8 1 466 472 2827008 10.1128/mcb.8.1.466-472.1988 PMC363152

[B55] Yamagishi M Ishida T Miyake A Cooper DA Kelleher AD Suzuki K Watanabe T Retroviral delivery of promoter-targeted shRNA induces long-term silencing of HIV-1 transcription Microbes Infect 2009 11 4 500 508 10.1016/j.micinf.2009.02.003 19233310

[B56] Barik S Site-directed mutagenesis by double polymerase chain reaction: megaprimer method Methods Mol Biol 1993 15 277 286 21400286 10.1385/0-89603-244-2:277

[B57] Yamochi T Yamochi T Aytac U Sato T Sato K Ohnuma K McKee KS Morimoto C Dang NH Regulation of p38 phosphorylation and topoisomerase IIalpha expression in the B-cell lymphoma line Jiyoye by CD26/dipeptidyl peptidase IV is associated with enhanced in vitro and in vivo sensitivity to doxorubicin Cancer Res 2005 65 5 1973 1983 10.1158/0008-5472.CAN-04-2611 15753397

[B58] Miyake A Ishida T Yamagishi M Hara T Umezawa K Watanabe T Horie R Inhibition of active HIV-1 replication by NF-kappaB inhibitor DHMEQ Microbes Infect 2010 12 5 400 408 10.1016/j.micinf.2010.02.004 20172044

[B59] Ishida T Hamano A Koiwa T Watanabe T 5' long terminal repeat (LTR)-selective methylation of latently infected HIV-1 provirus that is demethylated by reactivation signals Retrovirology 2006 3 69 10.1186/1742-4690-3-69 17034647 PMC1617119

[B60] Willey RL Smith DH Lasky LA Theodore TS Earl PL Moss B Capon DJ Martin MA In vitro mutagenesis identifies a region within the envelope gene of the human immunodeficiency virus that is critical for infectivity J Virol 1988 62 1 139 147 3257102 10.1128/jvi.62.1.139-147.1988 PMC250512

