# Supplementary figures and images for: HIV-1-encoded antisense RNA suppresses viral replication for a prolonged period
Source: Retrovirology. 2012 May 8;9:38. doi: 10.1186/1742-4690-9-38 (PMC3410806; doi:10.1186/1742-4690-9-38)

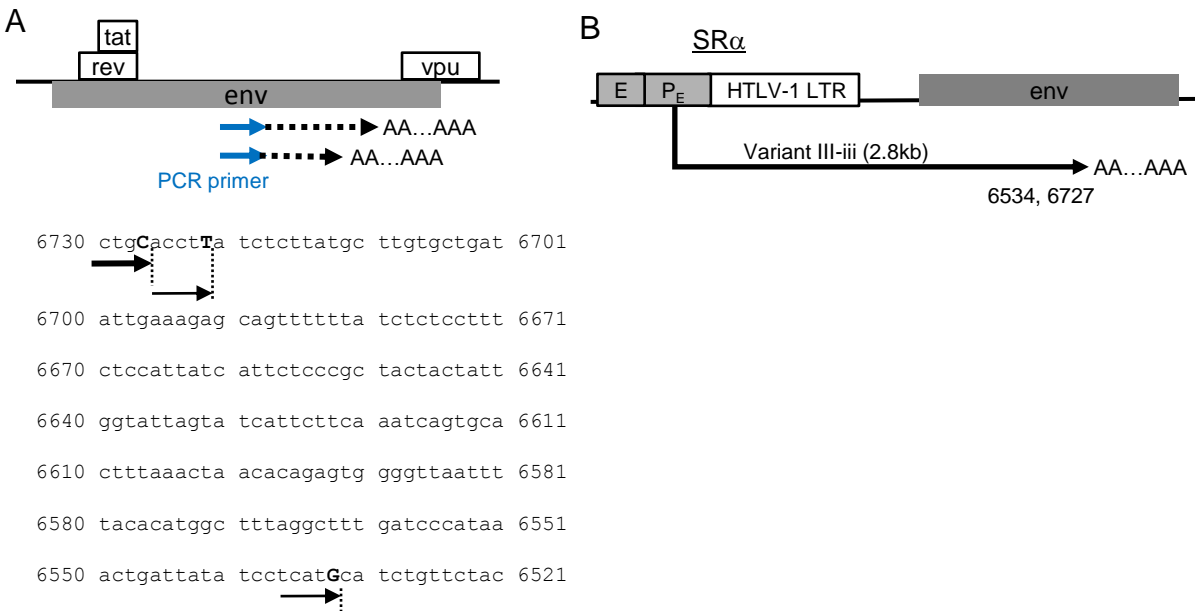

Supplement: Additional file 1: — Figure S1. Determination of transcript III-iii. (A) Results of 3' RACE. Top panel summarizes the results. Agarose gel electrophoresis of 3' RACE PCR products is shown in Figure 3B. The results of the sequence analyses are shown in the bottom panel. Bars and arrows indicate the identified termination sites. The bold arrow shows the major transcript. The upper case letters and arrows in the sequence indicate the termination sites of transcript III-iii. (B) Termination positions of transcript III-iii described in the context of pME18S-asHIV. [file 1742-4690-9-38-S1.pdf]

A

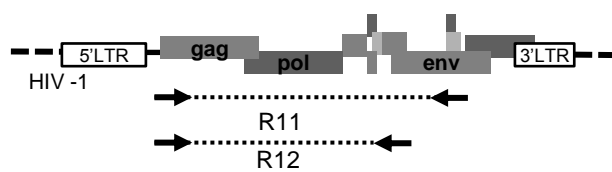

B

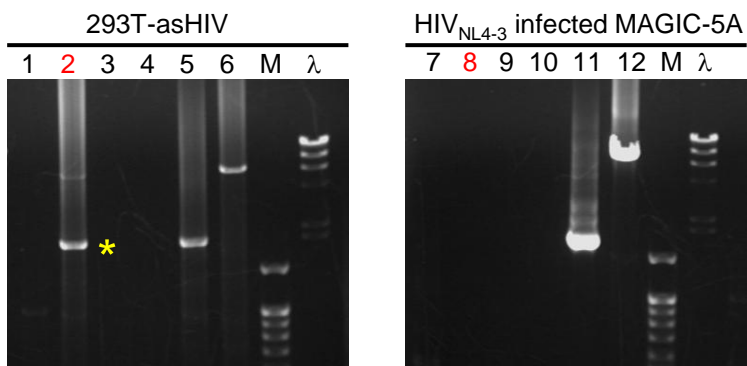

C

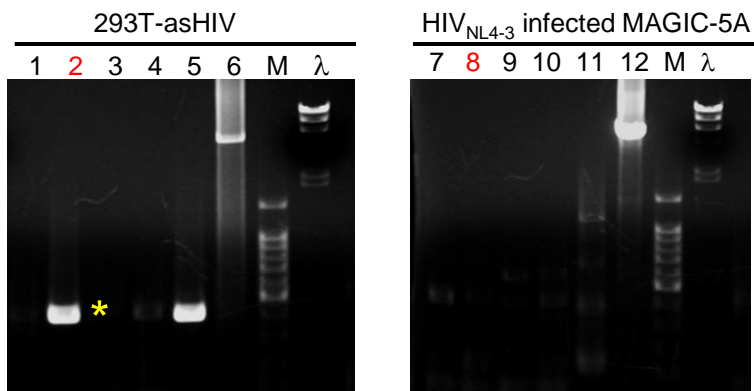

Supplement: Additional file 2: — Figure S2. Detection of spliced HIV-1 antisense RNAs. (A) A map of primer pairs at R11 and R12. (B-C) Results of agarose gel electrophoresis of antisense-specific RT-PCR products at R11 (B) and R12 (C). Expected PCR products derived from spliced transcripts were approximately 2 kb (B) and 400 bp (C), respectively (indicated by asterisks in lane 2), which are shorter than that of full-length (6 kb at R11 and 5 kb at R12). Experiments were performed using total RNAs from HEK293T with pME-18 S-asHIV (293 T-asHIV) (left panel) and HIV-1-infected MAGIC-5A (right panel). Lane 1 and 7, cells transfected with a mock vector or no infection control; Lane 2 and 8, cells with pME18S-asHIV or HIV-1 infection; Lane 3 and 9, no RTase control; Lane 4 and 10, no RT primer control; Lane 5 and 11, PCR products with conventional primer pairs with cDNA samples synthesized by random primers; Lane 6 and 12, positive control (amplified from pME18S-asHIV plasmid DNA, or from pNL4-3 plasmid DNA); M, 100 bp marker; Λ,Λ/Hind III marker. [file 1742-4690-9-38-S2.pdf]

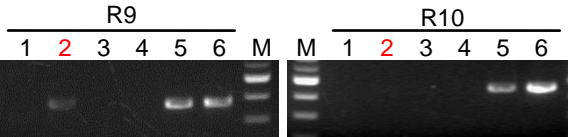

Supplement: Additional file 3: — Figure S3. HIV-1 antisense RNA pattern in infected cells. MAGIC-5A cells were infected with HIV-1NL4–3 and then analyzed antisense RNAs by antisense-specific RT-PCR at regions R9 (for original ASP mRNA) and R10 (for ASP-L). Representative results (n = 4) were shown. ASP-L was mainly detected. Lanes 1–6 are the same as in Figure 2B. [file 1742-4690-9-38-S3.pdf]

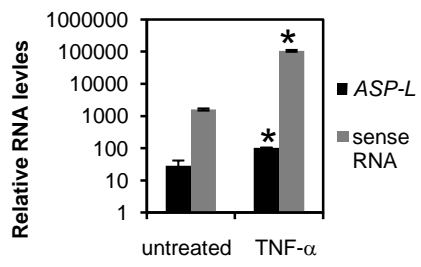

Supplement: Additional file 4: — Figure S4. Transcriptional activation of HIV-1 antisense RNAs by TNF-α treatment. ACH-2 was treated with TNF-α (10 ng/mL) for 24 h. Total RNAs were extracted and analyzed by strand-specific qRT-PCR at R7. The asterisks denote statistical significance relative to the untreated control (p < 0.02). [file 1742-4690-9-38-S4.pdf]

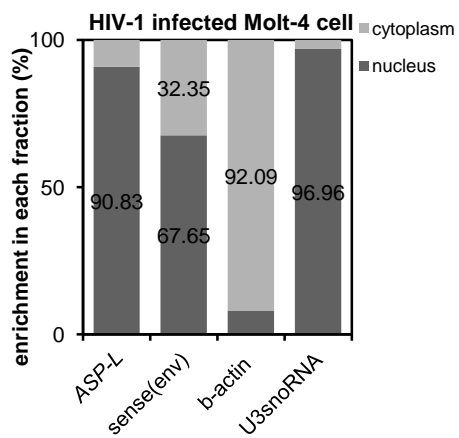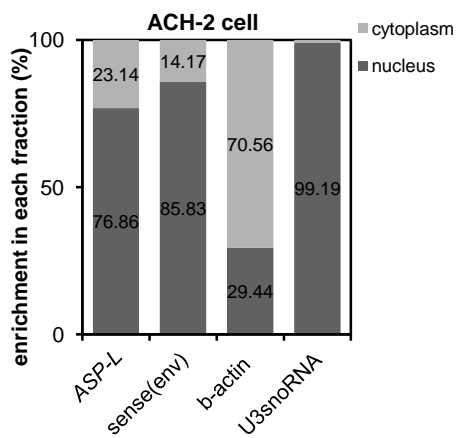

Supplement: Additional file 5: — Figure S5. Sub-cellular localization of HIV-1 antisense RNAs in the HIV-1 infected T cell lines. Results of subcellular localization analysis of T-cell lines. RNA samples were prepared from cytoplasmic and nuclear fractions of ACH-2 cells and HIV-1NL4–3-infected Molt-4 cells as described in the text. [file 1742-4690-9-38-S5.pdf]

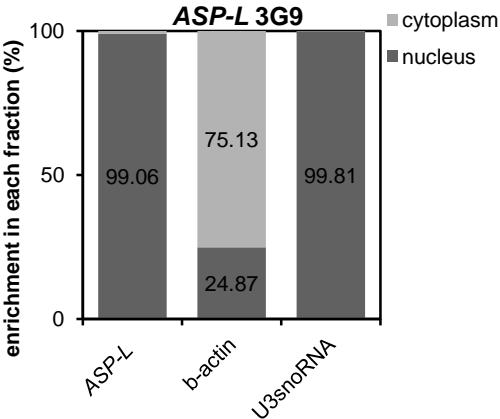

Supplement: Additional file 6: — Figure S6. Sub-cellular localization ofASP-Lin the Molt-4 cells stably expressingASP-L. RNA samples were prepared from cytoplasmic and nuclear fractions of Clone 3G9 cells. Results of the quantitative measurement of RT-PCR at R7 are presented from cDNAs synthesized with random primers. Fractionation efficiencies were confirmed by measuring the levels of β-actin cytoplasmic RNA and nuclear U3 snoRNA. [file 1742-4690-9-38-S6.pdf]

A

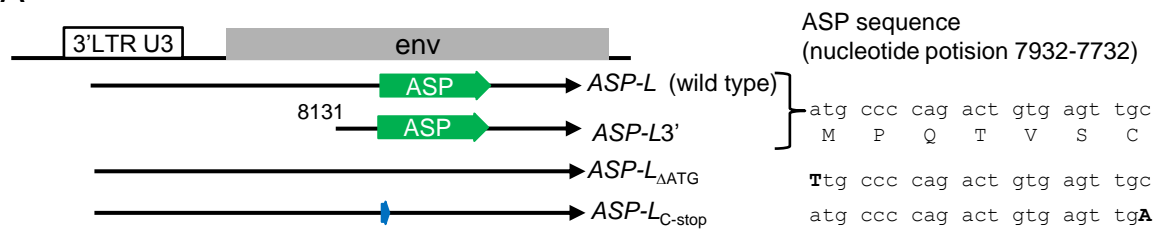

B

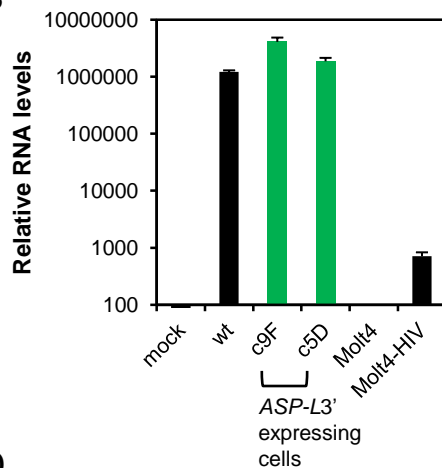

C

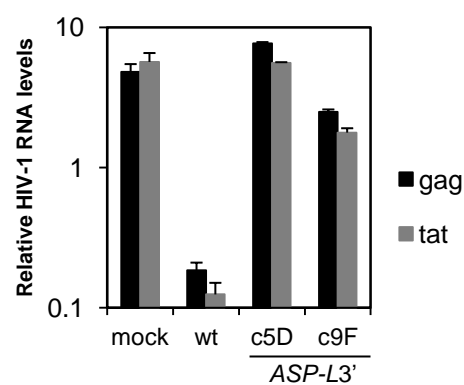

D

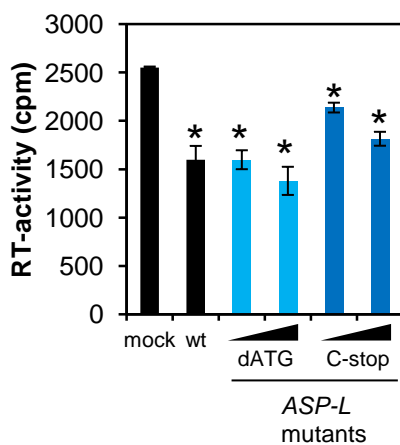

E

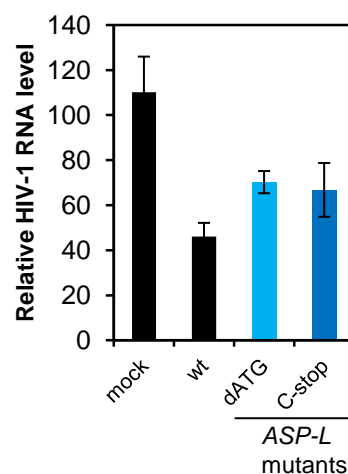

Supplement: Additional file 7: — Figure S7. Inhibitory effects of full-lengthASP-LRNA on HIV-1 replication. (A)ASP-L mutants. ASP-L3′ is a portion of ASP-L bearing the ASP-coding region. ASP-L∆ATG contains an A to T mutation at the start codon of ASP. ASP-LC-stop contains a C to A mutation at the seventh codon of ASP to convert Cysteine into a stop codon. Detailed sequences are provided in the right panels. Upper cases in the nucleotide sequences show the mutated sites. (B–C) Effects of ASP on HIV-1 replication. (B) Expression levels of ASP-L3′ measured by qRT-PCR at R7. mock, Clone 2B3 with the empty vector; wt, Clone 3C2 stably expressing wild type ASP-L (Figure 7D–G); c9F and c5D, established Molt-4 clones that stably express ASP-L3′; Molt4, uninfected Molt-4 cells; Molt4-HIV, HIV-1NL4–3 infected Molt-4. (C) HIV-1 RNA levels at 4 days post HIV-1NL4–3 infection. HIV-1 RNA levels were evaluated by qRT-PCR with gag and tat genes (mean ± S.D). (D) Effects of ASP-L RNA on HIV-1 replication. 50 ng or 200 ng of pIRES-RSV-ASP-L∆ATG (dATG) or pIRES-RSV-ASP-LC-stop (C-stop) was transfected into MAGIC-5A, followed by HIV-1NL4–3 infection. Viral production levels were evaluated by RT assays with the supernatants at 72 h post-transfection. (E) HIV-1 gag RNA levels at 48 h post-transfection measured by qRT-PCR.’mock’ stands for MAGIC-5A with empty vector. ‘wt’ stands for MAGIC-5A with pIRES-RSV-ASP-L. [file 1742-4690-9-38-S7.pdf]
