# Supplementary material for: HIV-1-encoded antisense RNA suppresses viral replication for a prolonged period
Source: Retrovirology. 2012 May 8;9:38. doi: 10.1186/1742-4690-9-38 (PMC3410806; doi:10.1186/1742-4690-9-38)
Supplement: Additional file 8: — Supplemental materials and methods. Supplemental materials and methods for expression vectors in Figure S7 are described. [file 1742-4690-9-38-S8.doc]

**Supplemental materials and methods**

The expression vector for *ASP-L*3’ was produced by removing the fragment (nucleotide position 8132 to 9451) by digestion with EcoRV/HindIII. The expression vector for *ASP-L* mutants, *ASP-L*_ΔATG_ and *ASP-L*_C-stop_, were produced by site-directed gene mutagenesis method [56, 57] using primers described in additional file 9.
